# Editorial: Improving outcomes in diabetic foot care - a worldwide perspective

**DOI:** 10.3389/fendo.2024.1505838

**Published:** 2024-10-23

**Authors:** Richard Paisey, José Luis Lázaro Martínez, Frances Game, Honda Hsu, Joanne Paton

**Affiliations:** ^1^ Research and Development, South Devon Healthcare National Health Service (NHS) Foundation Trust, Torquay, United Kingdom; ^2^ University Clinic of Podiatry, Complutense University of Madrid, Madrid, Madrid, Spain; ^3^ Department of Diabetes University Hospitals of Derby and Burton National Health Service (NHS) Foundation Trust, University Hospitals of Derby, Derby, United Kingdom; ^4^ Department of Medicine, Buddhist Tzu Chi General Hospital, Hualien City, Taiwan; ^5^ Faculty of Health, University of Plymouth, Plymouth, United Kingdom

**Keywords:** diabetic foot ulceration, wound care, offloading, tissue perfusion, exosome therapy, neurocutaneous skin flaps, low income countries

The International Diabetes Federation has documented the challenging increase in prevalence of diabetes mellitus now evident in virtually every country in the world (1). Over 90% have type 2 diabetes, and the majority of those living with diabetes live in low- or middle-income countries (2). The specific diabetes associated complications-retinopathy, nephropathy and neuropathy are compounded by the enhanced risk of atherosclerotic vascular disease. Peripheral neuropathy, peripheral vascular disease and susceptibility to infection result in a high incidence of diabetic foot disease manifested by foot deformity, ulceration, ischaemia and infection (3). The intractable nature of diabetic foot disease severely affects the quality of life and survival of those affected, impacts livelihood and family life and incurs enormous health care costs (4). The incidences and outcomes for diabetic foot disease are influenced by age, ethnicity, deprivation and availability of early diagnosis and treatments (5, 6).

In this Research Topic we have sought to collate a worldwide perspective to encourage sharing of differing approaches to diabetic foot care and cross-cultural debate. We have been privileged to receive manuscripts from high-, middle- and low-income countries, which have provided insights into- assessment of strategies to prevent and heal foot ulceration; risk factors for foot ulceration; morbidity and mortality; established and novel treatment options.

The key to prevention of diabetic foot wounds in high-risk subjects lies with patient engagement, and the group from Malaga assess the reliability and validity of a self- management questionnaires. The Birmingham group have shown that individuals with new-onset type 2 diabetes who had moderate to high risk of diabetic foot disease were more likely to die compared to those at low risk. Those who did not have foot examination had high risk both of foot ulceration and mortality. A review of wound healing from China offers hope that better preparation of exosomes could help healing in diabetic foot wounds. A metanalysis from India tackles the important issue of micronutrient deficiencies in Diabetic foot ulcer patients. Articles from Indigenous peoples in Canada and Nepal emphasize the need for a holistic approach to patient care and antimicrobial stewardship is evaluated in Peru. Dressings and debridement are reviewed in articles from Guizou in China and the intricacies of total contact casting versus removable casts and footwear in another metanalysis. From Chengdu in China another tackles the issue of standard versus advanced methods of debridement. Ozone therapy, platelet rich plasma application for DFU and micronutrient status in DFU are also presented. A single centre study from the UK highlights the value of national and local data analysis which has shown worse outcomes for diabetic foot disease in deprived populations during the Covid-19 epidemic. Finally, an important surgical report of distally based sural neurocutaneous flaps in severe foot wounds has shown considerable success with good healing and subsequent excellent patient mobility.

The scope of these articles is wide, highlighting the need for more insights from around the world, to share innovations to help reduce the incidence and improve outcomes in diabetic foot disease.
